# Lyme neuroborreliosis epidemiology in Sweden 2010 to 2014: clinical microbiology laboratories are a better data source than the hospital discharge diagnosis register

**DOI:** 10.2807/1560-7917.ES.2019.24.20.1800453

**Published:** 2019-05-16

**Authors:** Viktor Dahl, Karin T Wisell, Christian G Giske, Anders Tegnell, Anders Wallensten

**Affiliations:** 1 Public Health Agency of Sweden, Stockholm, Sweden; 2 European Programme for Intervention Epidemiology Training (EPIET), European Centre for Disease Prevention and Control (ECDC), Stockholm, Sweden; 3 Department of clinical microbiology, Karolinska University Hospital, Stockholm, Sweden.; 4 Division of microbiology, Department of Laboratory Medicine, Karolinska Institutet, Stockholm, Sweden; 5 Department of Medical Sciences, Uppsala University, Uppsala, Sweden

**Keywords:** **i**nfectious diseases, epidemiology, surveillance, borrelia, borreliosis, neuroborreliosis, Europe, Sweden, Lyme borreliosis, zoonosis, ticks, Ixodes

## Abstract

**Background:**

In a study from 2013 that prioritised communicable diseases for surveillance in Sweden, we identified Lyme borreliosis as one of the diseases with highest priority. In 2014, when the present study was designed, there were also plans to make neuroborreliosis notifiable within the European Union.

**Aim:**

We compared possibilities of surveillance of neuroborreliosis in Sweden through two different sources: the hospital discharge register and reporting from the clinical microbiology laboratories.

**Methods:**

We examined the validity of ICD-10 codes in the hospital discharge register by extracting personal identification numbers for all cases of neuroborreliosis, defined by a positive cerebrospinal fluid–serum anti-*Borrelia* antibody index, who were diagnosed at the largest clinical microbiology laboratory in Sweden during 2014. We conducted a retrospective observational study with a questionnaire sent to all clinical microbiology laboratories in Sweden requesting information on yearly number of cases, age group and sex for the period 2010 to 2014.

**Results:**

Among 150 neuroborreliosis cases, 67 (45%) had received the ICD-10 code A69.2 (Lyme borreliosis) in combination with G01.9 (meningitis in bacterial diseases classified elsewhere), the combination that the Swedish National Board of Health and Welfare recommends for neuroborreliosis. All 22 clinical laboratories replied to our questionnaire. Based on laboratory reporting, the annual incidence of neuroborreliosis in Sweden was 6.3 cases per 100,000 in 2014.

**Conclusion:**

The hospital discharge register was unsuitable for surveillance of neuroborreliosis, whereas laboratory-based reporting was a feasible alternative. In 2018, the European Commission included Lyme neuroborreliosis on the list of diseases under epidemiological surveillance.

## Introduction

Lyme borreliosis is a vector-borne bacterial zoonosis [1]. The vector is a tick of the family *Ixodes* and the reservoirs for the bacteria are mainly small animals such as birds or rodents that the ticks feed on [2]. Lyme borreliosis is caused by closely related species of bacteria commonly referred to as *Borrelia burgdorferi* sensu lato (Bb). Infection with Bb can clinically manifest as erythema migrans or later as a disseminated form such as lymphocytoma, chronic acrodermatitis, neuroborreliosis, arthritis and carditis [3].

The incidence of Lyme borreliosis is likely to differ across European countries, possibly depending on geographical and environmental factors affecting the presence of ticks, differences in the genotypes of *B. burgdorferi* occurring in different parts of Europe as well as differences in human behaviour influencing risk exposure. However, it is difficult to compare the incidence in different European countries owing to differences in case definitions and methods of data collection [4].

A survey in 2010 by the European Center for Disease Prevention and Control (ECDC) found that 23 of 28 responding European countries had surveillance systems in place for Lyme borreliosis, of which 16 were based on mandatory notifications [5]. Some countries had surveillance for erythema migrans, some for all disseminated forms of Lyme borreliosis and others only for Lyme neuroborreliosis [6,7].

In Sweden, Lyme borreliosis is not a mandatorily notifiable disease but some studies have been undertaken to estimate the incidence of the disease. In 1992 and 1993, all physicians working in counties in the south of Sweden were asked to report all cases of Lyme borreliosis regardless of the clinical manifestation. During that period, the annual incidence was 69 cases per 100,000 inhabitants [8]. Erythema migrans was the most common disease manifestation (77% of the cases) followed by Lyme neuroborreliosis (16% of the cases). A later study examining medical records from 1997 to 2003 estimated the annual incidence of erythema migrans at 464 cases per 100,000 in one of the counties (Blekinge) in south-eastern Sweden [9]. According to Swedish guidelines, the diagnosis of erythema migrans should be made without any laboratory confirmation, but the diagnosis of other clinical manifestations should be supported by serology; for Lyme neuroborreliosis, both cerebrospinal fluid (CSF) and serum should be analysed and an antibody index calculated to verify intrathecal antibody production. In addition to intrathecal antibody production, pleocytosis as a marker of inflammation is required for the diagnosis of Lyme neuroborreliosis [10,11].

In a study from 2013 that prioritised communicable diseases for surveillance according to their public health relevance in Sweden, we identified Lyme borreliosis as one of the diseases with the highest priority [12]. We therefore decided to explore the possibilities of surveillance for Lyme borreliosis in Sweden. The most important attributes for the surveillance system were simplicity, acceptability and stability. We decided that Lyme neuroborreliosis would be the most suitable disease manifestation to report because (i) the diagnosis is based on microbiological testing and therefore likely to be more specific than erythema migrans and (ii) Lyme neuroborreliosis was, at the time, likely to become notifiable within the European Union (EU) [4].

The hospital discharge diagnosis register as a data source met the requirements of a surveillance system for Lyme borreliosis. It is based on the ICD-10 codes given at discharge from inpatient stays and after outpatient visits, but these codes are not collected at the primary healthcare level [13]. Since the diagnosis of Lyme neuroborreliosis requires a lumbar puncture in order to calculate the antibody index, diagnosis and treatment occurs at hospitals and not in primary care, we did not anticipate that this would be a problem. However, the hospital discharge register does not use one single ICD-10 code for Lyme neuroborreliosis but rather different combinations of codes. The Swedish National Board of Health and Welfare recommends in the Swedish version of ICD-10 (called ICD-10SE) that the combination of A69.2 (Lyme borreliosis) and G01.9 (bacterial meningitis classified elsewhere) should be used, but to which extent this recommendation is followed is unknown. We were therefore concerned about the data quality in the hospital discharge register.

As an alternative data source we identified the clinical microbiology laboratories that perform the diagnostics for calculating the antibody index for Lyme borreliosis. Yearly collection of the number of cases through a questionnaire would also meet the criteria we had set up.

In this study, we aimed to validate the data in the hospital discharge register by studying which ICD-10 codes were used for Lyme neuroborreliosis in patients with a positive antibody index for Lyme borreliosis. We also explored the feasibility of laboratory-based surveillance by sending a questionnaire to all clinical microbiology laboratories in Sweden asking about the number of cases diagnosed for the years 2010 to 2014.

## Methods

### Validation of ICD-10 codes

In order to examine which ICD-10 codes were given to patients diagnosed with Lyme neuroborreliosis (including a positive antibody index), we extracted the personal identification numbers for all patients with a positive antibody index for whom samples had been analysed at the Karolinska University Hospital laboratory during 2014. The Karolinska University Hospital laboratory is the largest laboratory for clinical microbiology in Sweden, analysing samples mainly from the Stockholm region (ca 2.3 million inhabitants) but also from other regions. The list of personal identification numbers was sent to the Swedish National Board of Health and Welfare that maintains the hospital discharge register. This register is compiled yearly and contains the ICD-10 codes given to all patients in Sweden admitted to specialised inpatient care at a hospital or seen at a hospitals’ specialised outpatient clinics, i.e. it does not include the primary healthcare level. We received anonymised results containing all extracted ICD-10 codes given to the patients on our list, with the personal identification number replaced with a code but still containing data on sex and age. We calculated the proportion of patients who had received the ICD-10 code A69.2 for a diagnosis of borreliosis, and of these, how many had a code indicating disease in the central nervous system (G00–99).

### Questionnaire to clinical microbiology laboratories

We conducted a retrospective observational study by sending an online questionnaire to all clinical microbiology laboratories in Sweden asking if they performed diagnostics for Lyme neuroborreliosis. We asked laboratories conducting diagnostics about their catchment area and how many individuals with a positive antibody index for Lyme borreliosis they had observed between 2010 and 2014, including data on sex and age group for these individuals. We then calculated the national yearly incidence per 100,000, and by sex and age group for 2014. Laboratories not conducting diagnostics were asked to which laboratory they referred their samples.

### National data on ICD-10 code A69.2

We were concerned whether laboratory-based surveillance could provide reliable data on geographical distribution because of laboratories sending samples to each other and because the catchment area of the laboratories does not correspond to the NUTS3 level counties (Swedish: län). We therefore extracted the number of cases with the combination of ICD-10 codes that the Swedish National Board of Health and Welfare recommends should be used in Sweden for Lyme neuroborreliosis (A69.2 + G01.9) from the national hospital discharge register per county for the period 2010 to 2014 and calculated the yearly incidence rate per 100,000 per county. We then divided the incidence rate into 25 percentile groups and generated a choropleth map using ECDC’s online tool EMMa (https://emma.ecdc.europa.eu).

### Ethical considerations

We received clearance for this study from the regional ethical review board in Stockholm, Sweden (reference number: 2016–2125–21/2).

## Results

### Validation of ICD-10 codes

At the Karolinska University Hospital laboratory, 150 individuals were found to have a positive *Borrelia* antibody index in 2014 (**Table 1**). Of those, 84 (56%) were male. Their median age was 22 years (range: 2–89 years). We found that 67% (101/150) had been given the code for Lyme borreliosis (A69.2) and 52% (78/150) had it in combination with a code suggesting disease in the central nervous system (G00–G99). Forty-five per cent (67/150) had A69.2 in combination with G01.9 (meningitis in bacterial diseases classified elsewhere), the combination that the Swedish National Board of Health and Welfare recommends for Lyme neuroborreliosis.

**Table 1:** ICD-10 codes in the hospital discharge register for patients with a positive cerebrospinal fluid–serum antibody index for Lyme borreliosis, Stockholm, Sweden 2014 (n = 150)

	ICD-10 code	Number of cases	Percentage
**Borreliosis**	**A69.2**	**101**	**67**
Borreliosis in combination with			
Meningitis in bacterial diseases classified elsewhere	G01.9	67	45
Bell's palsy	G51.0	12	8
Other diseases of facial nerve, specified	G51.8	3	2
Other diseases of facial nerve, unspecified	G51.9	2	1
Clonic hemifacial spasm	G51.3	2	1
Polyneuropathy in diseases classified elsewhere	G63.0	2	1
Meningitis from other specified causes	G03.8	1	1
Meningitis unspecified	G03.9	1	1
Encephalitis, myelitis and encephalitis, unspecified	G04.9	1	1
Motor neuron disease	G12.2	1	1
Spastic hemiplegia	G81.1	1	1
Other reaction to spinal and lumbar puncture	G97.1	1	1
At least one of the above	G00–G99	78	52

### Questionnaire to clinical microbiology laboratories

All 22 clinical microbiology laboratories replied to our questionnaire. Seventeen of them performed diagnostics for Lyme neuroborreliosis. The remaining five laboratories sent their samples to one of the other Swedish laboratories who perform diagnostics.

Between 2010 and 2014, the number of cases of Lyme neuroborreliosis per year varied between 559 and 689. The year with the least cases was 2012 and the year with the most cases was 2011. (**Table 2**). Between 2010 and 2014, the number of cases per year varied between 559 and 689. The year with the least cases was 2012 and the year with the most cases was 2011. The incidence was highest in the age group of 0–9 year-olds, and in the age groups older than 50 years. (**Table 3**).

**Table 2:** Number of cases and incidence per year of patients with positive cerebrospinal fluid–serum antibody index for *Borrelia*, based on laboratory reporting, Sweden, 2010–2014 (n = 2,991)

	2010	2011	2012	2013	2014
Population	9,415,570	9,482,855	9,555,893	9,555,893	9,555,893
Cases	578	689	559	561	604
Incidence per 100,000	6.1	7.3	5.8	5.9	6.3

**Table 3:** Number of cases and incidence per age group of patients with positive cerebrospinal fluid–serum antibody index for *Borrelia*, based on laboratory reporting, Sweden, 2014 (n = 604)

Age (years)	Cases	Population	Incidence per 100,000
0–9	191	1,155,771	16.5
10–19	51	1,053,498	4.8
20–29	31	1,327,284	2.3
30–39	29	1,213,746	2.4
40–49	45	1,320,858	3.4
50–59	76	1,201,352	6.3
60–69	89	1,161,100	7.7
70–79	74	814,338	9.1
80–89	17	404,412	4.2
≥ 90	1	94,996	1.1
**Total**	**604**	**9,747,355**	**6.2**

### National data on ICD-10 code A69.2

All counties with the lowest incidence for Lyme neuroborreliosis were situated in the north of Sweden (Figure). The counties with the highest incidence were along the east and west coast of the southern part of Sweden and on the large islands in the Baltic Sea. 

**Figure:**
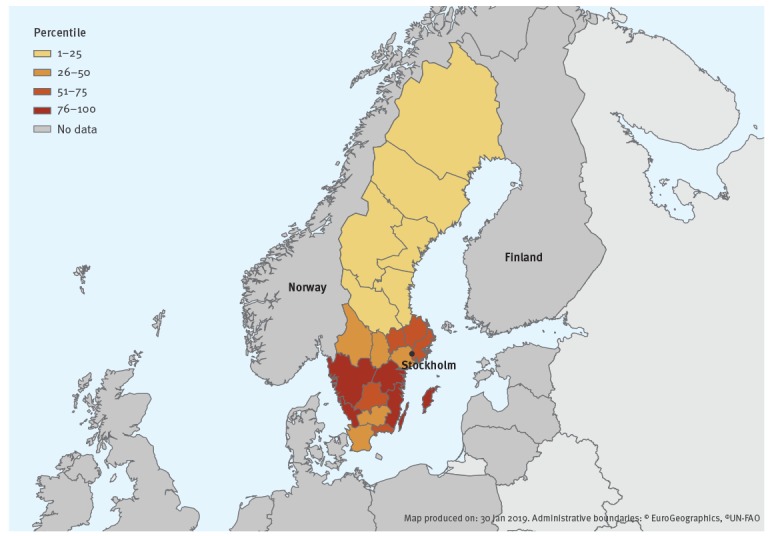
Annual incidence of Lyme neuroborreliosis (ICD-10 code A69.2 + G01.9), based on the hospital discharge register divided into 25 percentiles per county, Sweden, 2010–2014

## Discussion

In order to create a system for surveillance of Lyme neuroborreliosis in Sweden that would be simple, acceptable and stable, we evaluated two data sources: the hospital discharge register and the clinical microbiology laboratories. The Swedish National Board of Health and Welfare recommends the ICD-10 combination of G01.9 (Meningitis in bacterial disease classified elsewhere) and A69.2 (Lyme borreliosis) for Lyme neuroborreliosis but we were uncertain to which extent this recommendation was followed. Our validation confirmed our concern. Less than half of all patients with a positive CSF–serum antibody index for Lyme borreliosis were given the recommended combination of ICD-10 codes. Even when broadening the criteria and looking at Lyme borreliosis plus any code suggesting disease in the central nervous system, just above half of all patients were classified with such a combination. In fact, only two thirds received the code for Lyme borreliosis at all. To a small extent, this may be explained by the fact that some of these subjects did not have pleocytosis and/or neurological symptoms (i.e. did not meet the case definition for Lyme neuroborreliosis in the national guidelines). The low sensitivity for ICD-10 codes for Lyme neuroborreliosis found in our study suggest that the data in the hospital discharge register are unsuitable to base surveillance on.

Our finding is similar to what was observed in a study in Denmark where 1,047 of 3,000 (33%) patients with a positive CSF–serum antibody index for Lyme borreliosis also had a diagnosis of Lyme borreliosis in the Danish National Patient Registry (DNPR) [14].

We also evaluated the feasibility of laboratory-based surveillance. All laboratories that performed diagnostics for Lyme neuroborreliosis were able to provide data on the number of cases, including sex and age group of the cases, which suggest that this would be a feasible option for surveillance, even though it is an extra burden for the laboratories to extract data from the laboratory information management system and report the number of cases once a year. In the future, a national laboratory results database would be the best data source as the reporting could then be fully automatised.

The overall incidence for Lyme neuroborreliosis in Sweden was 6.3 per 100,000 for 2014. This was twice as high as the incidence rate in the neighbouring country Denmark which was 3.2 per 100,000 from 2010 to 2012 [6]. The Danish surveillance system also uses positive antibody index as the case definition, with the national microbiological database as the data source, so the data are comparable to our data. 

We found that the incidence was highest in children younger than 10 years; this is in agreement with a recent study on Lyme neuroborreliosis in children younger than 15 years in the south-western part of Sweden, where the incidence rate was lower in 10–14 year-olds compared with those younger than 10 years [15]. A similar age distribution with a higher incidence of Lyme neuroborreliosis in 5–10 year-old children and people older than 55 years was seen during an evaluation of the national surveillance system in Denmark [6].

The laboratories that did not perform Lyme borreliosis diagnostics sent their samples to other laboratories in Sweden. In some cases, the catchment area of a laboratory did not correspond to the NUTS3 level in Sweden (county, in Swedish: län). Therefore, we decided that it was not possible to use data from the clinical microbiology laboratories to describe the geographical distribution of Lyme neuroborreliosis in Sweden at NUTS3 level. If the reporting had been case-based with a personal identification number, instead of aggregated, we could have used national registries to determine the county of residence for each case to generate data on the geographical distribution. Instead, we used the combination of ICD-10 codes recommended by the Swedish National Board of Health and Welfare to calculate the yearly incidence per county. Based on our validation of ICD-10 codes, we did not believe it was an accurate measure of the incidence, but assuming that the error of coding did not vary too much between counties, we thought it could be useful to examine the geographical distribution. We found that the incidence was highest in the coastal regions in the south of Sweden. All counties with the lowest incidence were in the northern part of Sweden. This follows the distribution of ticks carrying Bb. These counties have, overall, a larger population and a higher population density which also could contribute to the higher incidence compared with the more northern counties. In 23 of 25 Swedish counties studied, the prevalence of Bb in ticks varied between 3 and 23%, with a higher prevalence in the southern counties [16]. This distribution may also reflect differences in climate as the northern parts of Sweden are cooler and ticks are only active when the temperature is above 4 °C [17].

The incidence of Lyme neuroborreliosis can be used to extrapolate the overall number of Lyme borreliosis cases. In a previous Swedish study, Lyme neuroborreliosis constituted 16% of the total incidence of Lyme borreliosis [8]. Assuming this proportion, the overall incidence for borreliosis in Sweden for 2014 would have been 39 cases per 100,000. This is higher than in Denmark where the average yearly incidence of Lyme neuroborreliosis was 3.2 per 100,000 from 2010 to 2012 and in Norway, where the yearly incidence of Lyme borreliosis was 4.7 cases per 100,000 from 1995 to 2013 [6,7]. It is on the same level as in Poland where an incidence of 35 cases per 100,000 was reported in 2015 [18], and lower than in Finland with an incidence of 120 per 100,000 in 2014 [19]. In 2006, the European countries with the highest estimated yearly incidence were Slovenia (206/100,000), Austria (135/100,000) and the Netherlands (103/100,000) [20]. Comparing the incidence between countries is difficult, however, given that surveillance systems (or the design of surveys for scientific purposes) and case definitions vary throughout Europe [20].

In 2018, the European Commission included Lyme neuroborreliosis on the list of diseases that are under epidemiological surveillance within the EU and a uniform EU case definition was released [21]. The European Centre for Disease Prevention and Control (ECDC) plans to start monitoring disease distribution in the EU and collect EU data in 2019.

Our study had several limitations. When validating the hospital discharge register, we only studied codes given to patients diagnosed at the Karolinska University Hospital laboratory in Stockholm, but coding practices may be different in different parts of Sweden. For our case definition (both for the validation of the hospital discharge register and for the data collection from the clinical microbiology laboratories), we included those with a positive antibody index but did not include criteria on pleocytosis and neurological symptoms which are required for Lyme neuroborreliosis diagnosis in clinical guidelines [22]. Since the antibody index is usually analysed at a clinical microbiology laboratory while the analyses to determine if pleocytosis is present are usually done at a laboratory for clinical chemistry, we had to disregard this aspect since it would be too time-consuming to check medical records for the presence of neurological symptoms and pleocytosis. Our approach would therefore lead to an overestimation of the number of Lyme neuroborreliosis cases because also those with a previous Lyme neuroborreliosis infection have a positive CSF–serum index (but no pleocytosis). However, because Lyme neuroborreliosis is uncommon, this effect is likely to be small. On the other hand, those with a possible early Lyme neuroborreliosis may not yet have developed a positive antibody index and were therefore not included, leading to an underestimation of the number of cases. In addition, we assumed that cases were infected in the county where the infection was diagnosed, which may not always have been the case.

## Conclusions and recommendations

We validated the hospital discharge diagnosis register and found that it is not a reliable source of data for Lyme neuroborreliosis surveillance. Laboratory-based surveillance is feasible and provides reliable data on the number of cases and their age and sex distribution, but fails to deliver reliable data on geographical distribution. Using laboratory data, we provide the first estimates of an incidence of Lyme neuroborreliosis in Sweden and show that it is higher in children than in adults.

We recommend that in Sweden, laboratory-based surveillance should be used for Lyme neuroborreliosis rather than surveillance based on ICD-10 codes. If possible, case-based reporting including a personal identification number should be used, so that the geographical distribution can also be estimated.

We recommend the implementation of a single code for Lyme neuroborreliosis when updating the Swedish version of ICD-10 to ICD-11 (in the English version, 1C1G.10 is used for Lyme neuroborreliosis) in order to facilitate coding and hopefully improving the data quality in the national hospital discharge diagnosis registry. The ICD-11 is currently being translated into Swedish by the National Board of Health and Welfare but it is not yet clear when it will be introduced. We suggest that other European countries validate the usage of codes before implementing surveillance based on ICD-10 codes.
